# EIF4A3-induced circular RNA SCAP facilitates tumorigenesis and progression of non-small-cell lung cancer via miR-7/SMAD2 signaling

**DOI:** 10.1007/s11356-023-26307-8

**Published:** 2023-04-20

**Authors:** Yingqing Zhang, Weibo Qi, Yonglei Wu

**Affiliations:** 1 Department of Respiratory, The First Hospital of Jiaxing (Affiliated Hospital of Jiaxing University), Jiaxing, 314000 Zhejiang People’s Republic of China; 2 Department of Cardiothoracic Surgery, The First Hospital of Jiaxing (Affiliated Hospital of Jiaxing University), Jiaxing, 314000 Zhejiang People’s Republic of China; 3 Jiaxing Key Laboratory of Precision Treatment for Lung Cancer, the First Hospital of Jiaxing (Affiliated Hospital of Jiaxing University), Jiaxing, 314000 Zhejiang People’s Republic of China

**Keywords:** NSCLC, Circular RNA SCAP, miR-7, Small mothers against decapentaplegic 2

## Abstract

The eukaryotic translation initiation factor 4A (eIF4A) family determines transcription efficiency by directly binding to precursor RNAs. One member, EIF4A3, modulates the expression of circRNAs. Circular RNA SCAP (circSCAP), a newly found circRNA, has been implicated in atherosclerosis. Yet, how circSCAP regulates cancer development and progression remains understudied. Here, we investigated the function of circSCAP and the molecular mechanism in the tumorigenesis and progression of non-small-cell lung cancer (NSCLC). CircSCAP was upregulated in both NSCLC tissues and cell lines and was mainly located in the cytoplasm. CircSCAP expression was promoted by EIF4A3, which was associated with poor prognosis in patients with NSCLC. CircSCAP sponged miR-7 to upregulate small mothers against decapentaplegic 2 (SMAD2). CircSCAP knockdown undermined cell proliferation, migration, and invasion abilities in NSCLC cell lines (SPCA1 and A549), which was rescued by either inhibiting miR-7 or overexpressing SMAD2. Moreover, circSCAP knockdown upregulated E-cadherin, while downregulating N-cadherin, Vimentin, and MMP9 in SPCA1 and A549 cells, which were abolished by either inhibiting miR-7 or overexpressing SMAD2. Additionally, miR-7 was markedly downregulated, whereas SMAD2 was significantly upregulated in NSCLC tissues. MiR-7 expression was inversely correlated with circSCAP and SMAD2 expression in NSCLC tissues. In conclusion, this study demonstrates that circSCAP is significantly upregulated in NSCLC cell lines and tissues and elucidates that circSCAP facilitates NSCLC progression by sponging miR-7 and upregulating SMAD2. The study provides a novel molecular target for early diagnosis and treatment of NSCLC.

## Introduction

Lung cancer is the most frequently diagnosed cancer in the respiratory system, causing the highest mortality and morbidity in patients with malignancy worldwide (Osmani et al. 2018; Siegel et al. 2021). Small-cell lung cancer (SCLC) and non-small-cell lung cancer (NSCLC) are two subtypes of lung cancer, accounting for 15% and 85% of diagnosed cases, respectively (Goldstraw et al. 2011). Although some advances in NSCLC treatment have been made in the last two decades, the overall survival rate of the patients remains low (Hirsch et al. 2017). Therefore, we need a better understanding of the molecular mechanism underlying the development and progression of NSCLC to improve the prognosis in patients with NSCLC.

Circular RNAs (circRNAs) are a novel class of non-coding RNA with a unique loop formed by back-splicing from a downstream 5′ site to an upstream 3′ site (Li et al. 2018a, b). CircRNAs are broadly expressed in mammalian cells with cell-specific expression patterns (Rybak-Wolf et al. 2015). CircRNAs can regulate various cell activities, such as proliferation, metastasis, and epithelial-mesenchymal transition (Zhu et al. 2020; Fan et al. 2021; Liu et al. 2021), which make them potent regulators in diverse disorders, such as diabetic nephropathy, cardiovascular diseases, pulmonary hypertension, and atherosclerosis (Li et al. 2018a, b; Hong et al. 2021; Wang et al. 2021; Wang and Bai 2021). Therefore, circRNAs are considered as promising clinical biomarkers and therapeutic targets for cancer diagnosis and treatment. Moreover, the circRNA-miRNA-mRNA signaling axis has been implicated in NSCLC development and progression. For instance, the circ-CPA4/let-7/PD-L1 axis regulates NSCLC cell growth and stemness, immune evasion, and drug resistance (Hong et al. 2020); circFGFR1 promotes anti-PD-1 resistance by sponging miR-381-3p in NSCLC cells (Zhang et al. 2019a, b); and circTP63 facilitates the tumorigenesis and progression of LUSC by regulating the miR-873-3p/FOXM1 signaling (Cheng et al. 2019). Circular RNA SCAP (circSCAP) is a newly identified circRNA and can promote NSCLC progression by activating p53 signaling through interacting with SF3A3 (Chen et al. 2022). In addition, it aggravates macrophage injury in atherosclerosis (He et al. 2021). Despite that, our understanding of the function and mechanism of circSCAP as competing endogenous RNAs (ceRNAs) in NSCLC is limited.

MicroRNAs (miRNAs) typically act as a downstream target of circRNAs, playing multifaceted roles in a wide range of diseases. For example, miR-7 is associated with the progression of diverse malignant tumors, such as gastric cancer (Ye et al. 2019), ovarian cancer (Li et al. 2019), and breast cancer (Huang et al. 2019). Interestingly, miR-7 appears to be versatile in lung cancer progression by modulating disparate signaling pathways. It can inhibit cell proliferation, migration, and invasion in NSCLC cells by targeting FAK through the ERK/MAPK signaling pathway (Cao et al. 2016). The miR-7/TGF-b2 axis sustains lung cancer metastasis induced by the acidic tumor microenvironment (Su et al. 2022). In addition, upregulation of miR-7 by EGFR through the Ras/ERK/Myc signaling promotes lung tumorigenesis (Chou et al. 2010). Moreover, the miR‑7/SP1/TP53BP1 axis is necessary for NSCLC radiosensitivity (Guo et al. 2020). Accordingly, we speculate that circSCAP could sponge miR-7 to regulate NSCLC development and progression.

This study investigated the function of circSCAP and elucidated the related molecular mechanism in NSCLC progression, demonstrating the importance of circSCAP in NSCLC progression and providing a potential therapeutic strategy for NSCLC.

## Materials and methods

### Clinical tissue samples

In total, 68 pairs of NSCLC tissue and adjacent normal tissue specimens were surgically dissected from the patients at The First Hospital of Jiaxing from June 2016 to October 2020. The patients had not received any radiotherapy, chemotherapy, targeted therapy, or immunotherapy before surgery. Cancer and non-cancerous regions were confirmed by two pathologists. Paired adjacent non-tumor tissue samples were taken from an area 5 cm away from the NSCLC focus and were negative for tumor cells. All collected specimens were immediately snap-frozen in liquid nitrogen (Delun, Shanghai, China) and stored at − 80 °C before RNA isolation. The detailed clinicopathological features are described in Table 1. This study was authorized by the Institutional Ethics Committee of The First Hospital of Jiaxing. Informed consent was obtained from all enrolled patients. The study abided by the Declaration of Helsinki (2016).

**Table 1:** Clinicopathological data of the 68 NSCLC patients

Variable	circSCAP expression		*P* value
	Low (34)	High (34)	
Age			
< 60	14	18	0.331
≥ 60	20	16	
Gender			
Male	16	17	0.808
Female	18	17	
Tumor size (cm)			
< 3	23	13	0.015*
≥3	11	21	
Differentiation			
Well	25	16	0.026*
Moderate-poor	9	18	
Lymph node metastasis			
Negative	22	13	0.029*
Positive	12	21	
Smoking			
Yes	16	22	0.143
No	18	12	
Distance metastasis			
* M*_0_	22	13	0.029*
* M*_1_	12	21	

*p<0.05

### Cell culture and transfection

The normal human bronchial epithelial cell line (16HBE) and four NSCLC cell lines (SPCA1, A549, CALU3, and H1229) (ATCC, USA) were cultured under 5% CO_2_ at 37 °C in DMEM medium (Hyclone, USA) supplemented with 20% fetal bovine serum (FBS, Gibco, USA). For cell transfection, siRNAs against circSCAP (si-circSCAP, 5′‐GGCGGCTACCCACTGCTGAAA‐3′) and EIF4A3 (si-EIF4A3, 5′‐AAUCAUAUCAAAAACACGCCC‐3′), negative control (si-NC), and pcDNA3.1 ( +) circRNA vector for overexpressing EIF4A3 (OE-EIF4A3) and SMAD2 (OE-SMAD2) were custom synthesized by GeneChem Company (GeneChem, Shanghai, China). MiR-mimics, miR-7 mimics, miR-inhibitor, and miR-7 inhibitor were obtained from RiboBio Company (RiboBio, Guangzhou, China). When cell confluence reached about 70%, Lipofectamine 3000 reagent (Sigma Aldrich, USA) was applied to transfect cells according to the supplier’s procedures (Chen et al. 2021).

### Bioinformatics analysis

Gene Expression Omnibus (GEO) database (https://www.ncbi.nlm.nih.gov/geo/) was used to screen the dysregulated circRNAs in NSCLC. CircInteractome (https://circinteractome.irp.nia.nih.gov/circular_rna.html) was employed to explore the interaction between circSCAP and miRNA. TargetScan (Release 7.1, http://www.targetscan.org) was used to identify the miRNA targeting site located in the 3′-UTR of SMAD2 (Wang et al. 2018a, b).

### Quantitative real-time PCR

Total RNAs were isolated from NSCLC tissues and cell lines with TRIzol reagent (Sigma Aldrich, USA). Mature miRNAs were quantified using the TaqMan MicroRNA assay (Life Technologies, USA). cDNA was obtained from 500 ng of total RNAs by reverse transcription, and the PCR was performed using the SYBR Green Master Mix Kit (Takara, Japan) following the supplier’s procedures. The circRNA and mRNA levels were normalized to *GAPDH*, and the miRNA level to *U6*. PCR conditions were as follows: pre-denature at 95 °C for 2 min; followed by 35 cycles of denaturation for 15 s at 95 °C, annealing at 60 °C for 20 s, and elongation at 72 °C for 10 s; and finally post-elongation at 72 °C for 2 min. The relative gene expression levels were determined by the 2^−ΔΔCt^ method (Zhang et al. 2021). The qRT-PCR primers were listed in Table 2.

**Table 2:** Primers used for qPCR

Gene (accession)	Forward 5′-3′	Reverse 5′-3′
circSCAP (NC_000001)	5′-CGAGGGACAGCAGTCAGAACA-3′	5′-GTGGCGGAGTCTTCCCTTATT-3′
miR-7 (NT_033778)	5′-AGACAGATAGCCCGCAGAGG-3′	5′-GATCTGCTGCCCTTGTGCTGTC-3′
SMAD2 (NG_029946)	5′-TGGTCTATGTCCGGTCCCATG-3′	5′-GCTGATTTGTGGGTGTGGAA-3′
U6 (NC_015438)	5′-CGCTTCCAGCACATATAC-3′	5′-CGCTTCGGCAGCACATATAC-3′
GAPDH (NG_007073)	5′-GCGGGGGAGCAAAGGGT-3′	5′-TGGGTGGCAGTGATGGCATGG-3′

### Western blot

Crude proteins were isolated from NSCLC cells following a previously reported protocol (Wei et al. 2020). Briefly, the cells were first treated with ice-cold RIPA lysis buffer (Sigma, USA) for 15 min and then boiled for 10 min to release proteins. The denatured protein (25 μg) was loaded into 12% SDS-PAGE. Following electrophoresis, the separated proteins were transferred onto a polyvinylidene difluoride (PVDF) membrane (Millipore, USA). Next, the membrane was blocked in 5% non-fat milk, followed by incubation with primary antibodies against N-cadherin (1:1000, CST, USA), EIF4A3 (1:2000, Abcam, USA), E-cadherin (1:2000, Abcam, USA), MMP9 (1:2000, Abcam, USA), Vimentin (1:2000, Abcam, USA), and GAPDH (1:2000, Proteintech, USA) overnight at 4 °C. Next, the membrane was treated with the secondary antibody (1:10,000, Jackson, USA) for 2 h at room temperature. Finally, ECL reagent (Amersham, UK) was applied to detect the protein bands. The grey value of each band was assessed with ImageJ software (National Institutes of Health, USA) (Chen et al. 2020).

### Subcellular fractionation

Nuclear and cytosolic fractions of NSCLC cells were isolated using a Cytoplasmic Nuclear RNA Purification Kit (Norgen Biotek Corp., Canada) following the supplier’s procedures. Further, qRT-PCR was performed to detect circSCAP in the cDNA of the nuclear and cytosolic fractions (Xie et al. 2022). *U6* and *GAPDH* served as the controls for transcript quantification in nuclear and cytoplasmic samples, respectively.

### Actinomycin D and RNase R treatment assay

To check circRNA stability and mRNA integrity, cells were treated with Actinomycin D (1 μg/mL) for 0, 6, 12, and 24 h to block RNA transcription. Then, RNAs were collected by the TRIzol reagent (Invitrogen, USA) for qRT-PCR analysis. For the RNase R assay, 2 μg of total RNAs from NSCLC cell lines was incubated with or without RNase R (3 U/mg; Epicentre Technologies, USA) for 15 min at 37 °C. Subsequently, the RNA was purified by the RNeasy MinElute Cleaning Kit (Qiagen) for qRT-PCR analysis (Li et al. 2021a, b).

### Dual-luciferase reporter assay

The day before transfection, a total of 5 × 10^4^ cells were seeded in a 24‐well plate. Luciferase reporter vectors, including circSCAP wild-type (circSCAP-WT), circSCAP mutant (circSCAP-Mut), SMAD2 wild-type (SMAD2-WT), and SMAD2 mutant (SMAD2-Mut), were subcloned in the pGL3-basic plasmid (Promega, USA) and were co-transfected with mimic control or miR-7 mimics using the Lipofectamine 3000 regents (Thermo Fisher Scientific, USA). After transfection for 72 h, transfected cells were harvested, and the luciferase activity was measured by the Luciferase Reporter Assay System (Promega, USA). Each experiment was repeated three times (Bing et al. 2021).

### RNA immunoprecipitation

EZ Magna RIP Kit (Millipore) was used for RNA immunoprecipitation (RIP) following the manufacturer’s instructions. Briefly, cells were lysed in complete RIP lysis buffer. Then the cell lysates were incubated with magnetic beads conjugated with an anti‑Ago2 antibody (1:2000, Abcam, USA) or a normal IgG antibody (1:2000, Abcam, USA) for 24 h at 4 °C. After that, the magnetic beads were washed and treated with proteinase K (50 μg/mL) at 37 °C for 1 h to remove protein. Finally, circSCAP, miR-7, and SMAD2 in immunoprecipitated RNAs were quantified by qRT‐PCR analysis (Qin et al. 2021).

### RNA pull-down assay

Biotin-labeled probes against circSCAP were designed and synthesized by Sangon Biotech (Shanghai, China). The interaction between circSCAP and miR-7 in NSCLC cells was verified by the RNA pull-down assay. In brief, the cells were transfected with bio-circSCAP by Lipofectamine 3000 (Thermo Fisher Scientific, USA) following the supplier’s procedures. After transfection for 48 h, the cells were harvested and lysed. The cell lysates were incubated with magnetic beads (Life Technologies, USA). After three washes with 1 × PBS, the expression level of miR-7 was determined by qRT‐PCR (Hong et al. 2020).

### CCK‑8 assay

Cell proliferation capability was assessed by the CCK‑8 assay. Briefly, the cells were transfected with respective constructs for 72 h. The transfected cells (1 × 10^4^ cells/well) were then seeded into 96‑well plates followed by further incubation for 0 h, 24 h, 48 h, and 72 h. Lastly, 10 μL of CCK‑8 reagent (Beyotime, Shanghai, China) was applied to each well and further incubated at 37 °C for 2 h. Finally, a microplate reader (Bio-Rad Laboratories, USA) was used to detect the absorbance at 450 nm wavelength. All experiments were performed in triplicate (Luo et al. 2021).

### Wound healing assay

Cell migration capability was measured by the wound healing assay. First, the cells (5 × 10^5^/well) were seeded into 6-well plates and grown to reach 80–90% confluence. A blue pipette (1 mL) was used to scratch to create a linear wound, and then serum-free DMEM medium was added followed by different treatments for another 24 h. The images were taken at 0 and 24 h under an inverted microscope (TS100-F, Japan), and ImageJ software was used to measure the wound area (Zhang et al. 2020a, b).

### Transwell assay

Cell metastasis capability was measured using Matrigel-coated transwell plates (BD Biosciences, USA). First, the cells were transfected with indicated constructs for 72 h. Then 1 × 10^4^ transfected cells were suspended in serum-free medium and applied to the upper compartment, and culture medium containing 10% FBS was applied to the lower compartment. After incubation for 24 h, the remaining cells in the upper compartments were removed by cotton swabs, and the cells that penetrated through the membrane were stained with 1.5% crystal violet (Solarbio, Beijing, China) at 37 °C. The invaded cells were observed under an inverted microscope (Leica, Germany). Three biological replications were included in each experiment (Xie et al. 2022).

### Statistical analyses

All experimental data were analyzed by Graph Prism 7.0 (GraphPad Software, San Diego, CA, USA) and were presented as mean ± SD. The significance between two groups was determined by two-tailed Student’s *t*-test, and that among multiple groups by one-way ANOVA followed by Holm-Sidak multiple comparisons. The survival curve was established using the Kaplan–Meier plot. The correlations between the expression levels of circSCAP, miR-7, and SMAD2 in NSCLC tissues were analyzed by the Pearson *χ*^2^ test. The level of significance was set as* P* < 0.05.

## Results

### CircSCAP is upregulated in NSCLC tissues and cell lines

**Fig. 1:**
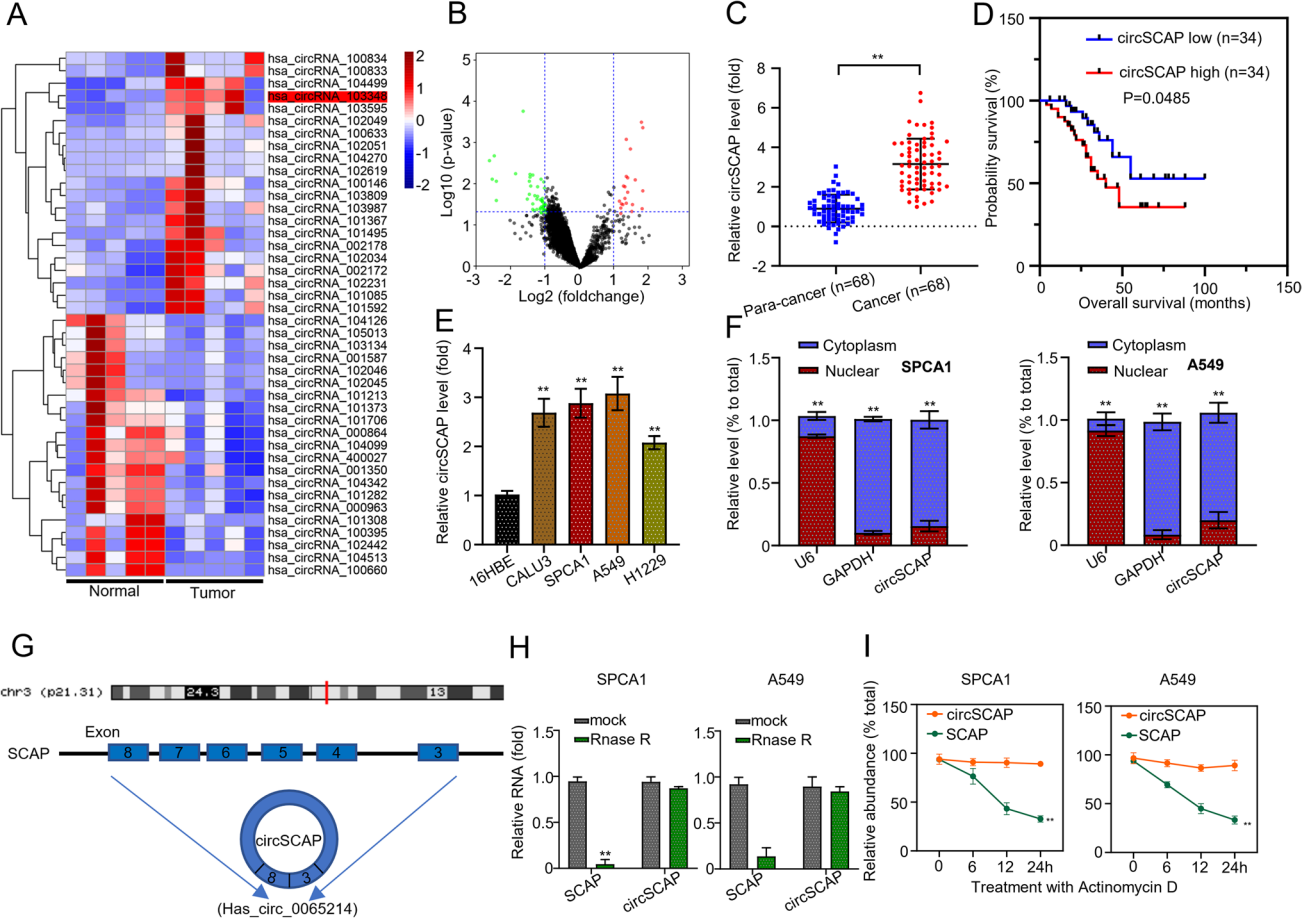
circSCAP is elevated in both NSCLC tissues and cell lines. **A** Expression levels of circSCAP in 5 pairs of NSCLC tissues and adjacent non-cancerous specimens in the GEO database. **B** Volcano plot of differentially expressed circRNAs in 5 pairs of NSCLC tissues and adjacent non-cancerous specimens in the GEO database. **C** Expression levels of circSCAP in 68 pairs of NSCLC tissues and adjacent non-cancerous specimens were calculated by qRT‐PCR. **D** Kaplan-Meier plot of the survival of NSCLC patients with low or high circSCAP expression. **E** Expression levels of circSCAP in the human bronchial epithelial cell line (16HBE) and the four NSCLC cell lines (SPCA1, A549, CALU3, and H1229) were calculated by qRT‐PCR. **F** Subcellular location of circSCAP in SPCA1 and A549 cell lines was determined using cell cytosolic/nuclear fraction assay. **G** Schematic illustration of circSCAP formation by back splicing. **H** Expression levels of circSCAP and SCAP inSPCA1 and A549 cell lines with or without RNase R treatment were calculated by qRT‐PCR. **I** Expression levels of circSCAP and SCAP inSPCA1 and A549 cell lines with Actinomycin D treatment were calculated by qRT‐PCR. All experiments were performed in triplicate. *P < 0.05, **P < 0.01

To screen the dysregulated circRNAs in NSCLC, we downloaded the circRNA expression profiles from the Gene Expression Omnibus (GEO) database (https://www.ncbi.nlm.nih.gov/geo/). In total, we identified 21 upregulated and 47 downregulated circRNAs in NSCLC tissues (*n* = 5) relative to adjacent non-cancerous specimens (*n* = 5) (Fig. 1A). Among them, circSCAP was significantly upregulated (Fig. 1B). To verify the upregulation of circSCAP in NSCLC, we quantified circSCAP transcripts in 68 pairs of NSCLC tissues and adjacent non-cancerous specimens. qRT‐PCR showed that the expression level of circSCAP was significantly higher in the NSCLC tissues than in the adjacent non-cancerous specimens (Fig. 1C). Next, to determine whether circSCAP upregulation was correlated with the survival rate, the 68 NSCLC patients were divided into two groups (the high- and the low-expression groups) based on the median expression value of circSCAP. The Kaplan–Meier analysis clearly showed that the circSCAP expression was inversely correlated with the overall survival rate of NSCLC patients (Fig. 1D). Similarly, the expression level of circSCAP was positively correlated with tumor size, cell differentiation, lymph node metastasis, and distant metastasis in NSCLC patients (Table 1). Next, we detected the differential expression of circSCAP in the human bronchial epithelial cells line (16HBE) and four NSCLC cell lines (SPCA1, A549, CALU3, and H1229). CircSCAP expression in the four NSCLC cell lines was upregulated, with SPCA1 and A549 cells exhibiting the highest CircSCAP level (Fig. 1E). Next, we determined the subcellular localization of circSCAP in SPCA1 and A549 cells using the cell cytosolic/nuclear fractions assay. CircSCAP was mainly located in the cytoplasm (Fig. 1F), implicating circSCAP in NSCLC progression.

Figure 1G shows the formation of circSCAP by back-splicing from the *SCAP* locus 47466974–47476627 on chromosome 3 (Fig. 1G). Next, we verified the stability of circSCAP by RNase R and Actinomycin D treatment. Linear SCAP was digested by RNase R, whereas circSCAP was not (Fig. 1H). Similarly, circSCAP was refractory to SCAP treatment, while linear SCAP was not (Fig. 1I). Collectively, these data demonstrate the circular nature of circSCAP.

### EIF4A3 promotes circSCAP expression

**Fig. 2:**
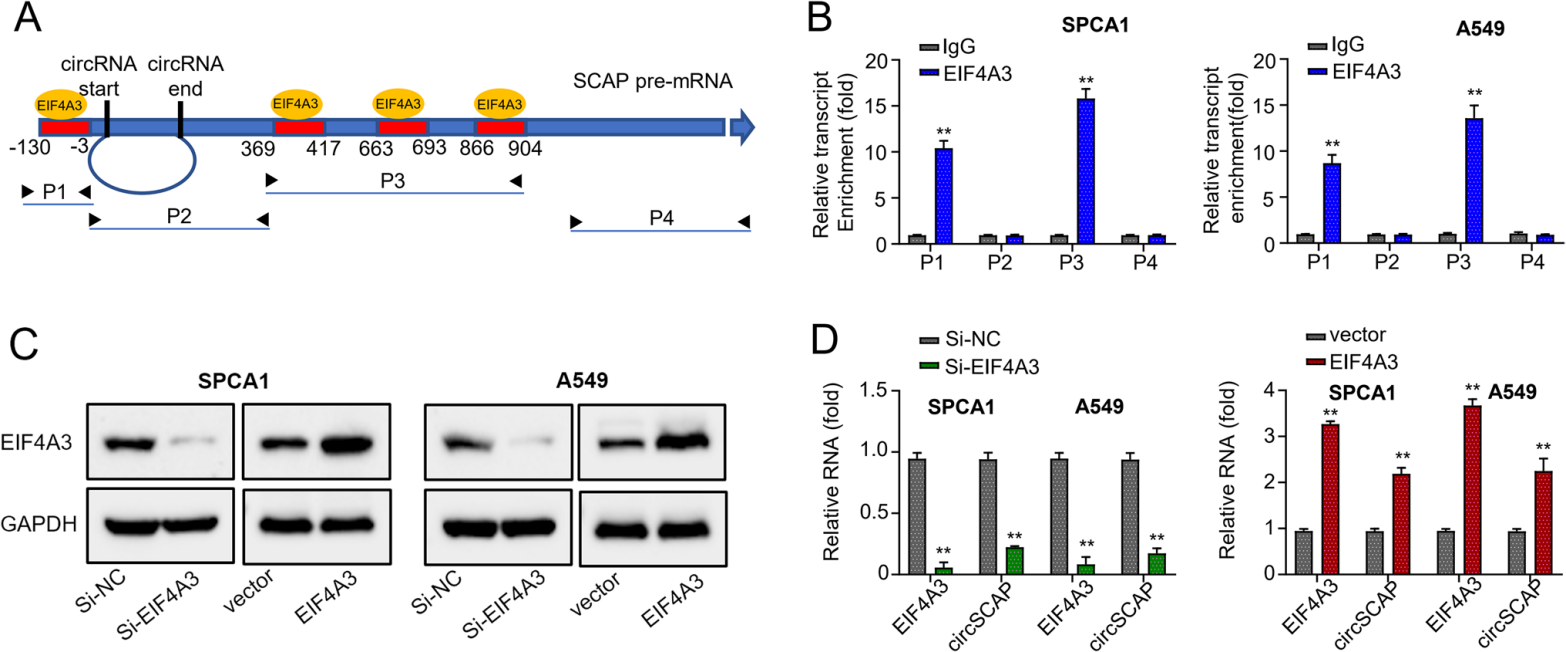
EIF4A3 promotes circSCAP expression. **A** Schematic representation of the EIF4A3 binding site in the upstream of circSCAP pre mRNA. **B** Binding of EIF4A3 to circSCAP was confirmed by RNA immunoprecipitation (RIP) assay. **C** Protein levels of EIF4A3 in SPCA1 and A549 cells transfected with OE-vector or OE-EIF4A3 plasmids and si-NC or si-EIF4A3 were determined by Western blot. **D** Expression levels of circSCAP inSPCA1 and A549 cells transfected with OE-vector or OE-EIF4A3 plasmids and si-NC or si-EIF4A3 were determined by qRT‐PCR. All experiments were performed in triplicate. *P < 0.05, **P < 0.01

Next, we set out to identify the factor that promotes circSCAP expression in NSCLC cell lines. First, we screened the CircInteractome database (https://circinteractome.nia.nih.gov/) and identified a putative binding site for EIF4A3 in the upstream of circSCAP precursor mRNA (Fig. 2A). Then, we performed the RIP assay to confirm the interaction between EIF4A3 and circSCAP. Two SCAP pre-mRNA segments, P1 and P3, harboring the putative EIF4A3 binding site were enriched with EIF4A3, while P2 and P4 were not (Fig. 2B). Furthermore, EIF4A3 was silenced or overexpressed in SPCA1 and A549 cells through transfection with si-EIF4A3 or OE-EIF4A3 plasmids. Both mRNA and protein levels of circSCAP were diminished when EIF4A3 was silenced but were elevated when EIF4A3 was overexpressed in these two cell lines (Fig. 2C and D), suggesting that EIF4A3 promotes circSCAP expression by direct binding to the upstream of SCAP pre-mRNA.

### CircSCAP knockdown represses the proliferation and metastasis ability of NSCLC cells *in vitro*

**Fig. 3:**
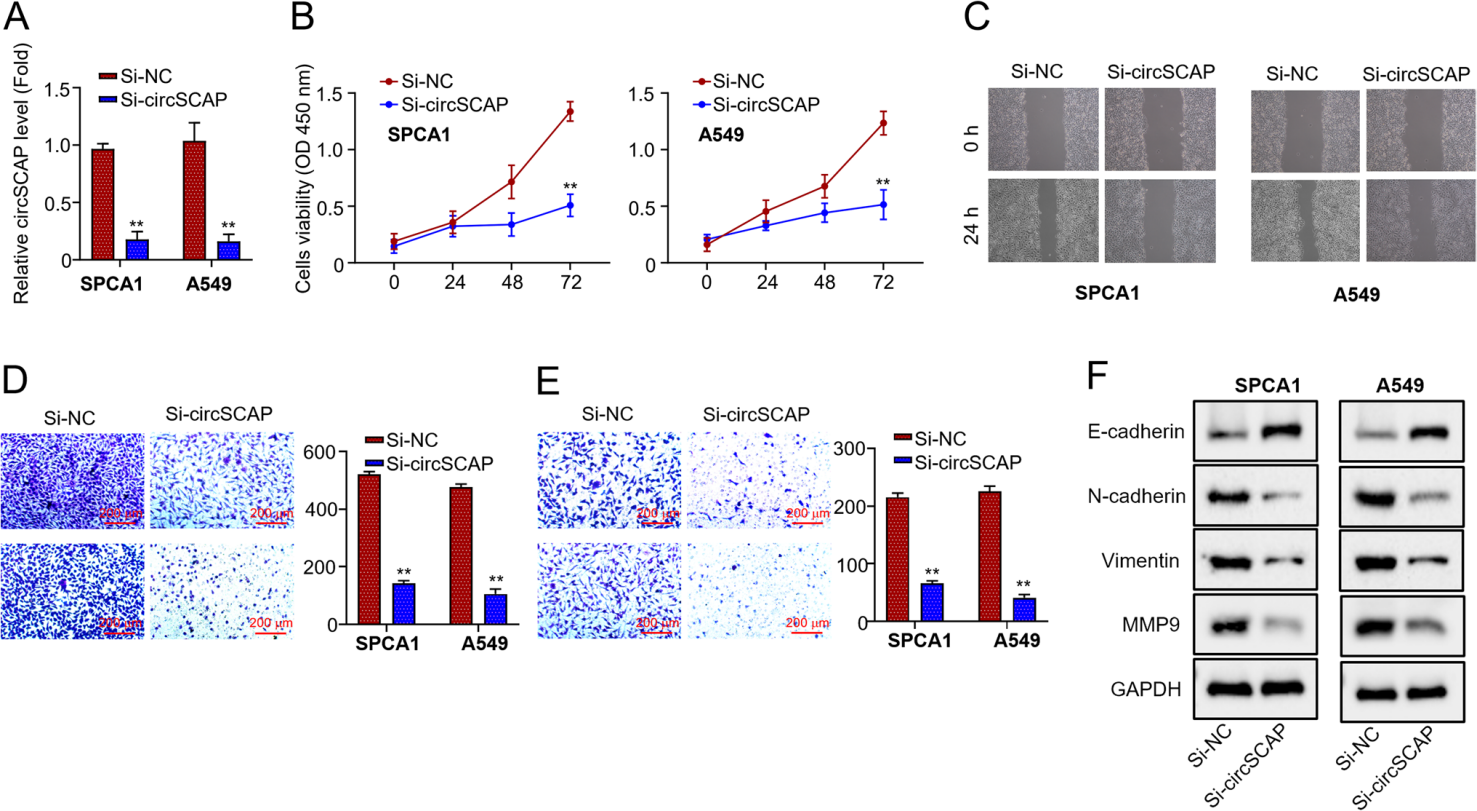
circSCAP knockdown represses the NSCLC cell proliferation and metastasis ability in vitro. **A** Expression levels of circSCAP inSPCA1 and A549 cells transfected with si-NC or si-circSCAP were calculated by qRT‐PCR. **B** Cell proliferation in (A) was determined through CCK-8 assay. **C** Number of migrated cells in (A) was calculated by wound healing assay. **D** Cell migration capability in (A) was assessed by transwell assay (uncoated with Matrigel). **E** Cell invasion capability in (A) was measured by transwell assay (coated with Matrigel). **F** Protein levels of E-cadherin, N-cadherin, Vimentin, and MMP9 in (A) were determined by Western blot. All experiments were performed in triplicate. *P < 0.05, **P < 0.01

To functionally dissect circSCAP in NSCLC carcinogenesis and progression, we knocked down circSCAP inSPCA1 and A549 cells by transfection with two si-RNAs, si-circSCAP #1 and si-circSCAP #2 (Fig. 3A). Then we performed the CCK-8 assay to determine the cell proliferation ability. We found that circSCAP knockdown potently suppressed cell proliferation (Fig. 3B). Moreover, the wound healing assay indicated that circSCAP knockdown decreased the number of migrated cells (Fig. 3C), and the transwell assay showed that circSCAP knockdown impaired the cell migration and invasion of SPCA1 and A549 cell lines (Fig. 3D and E). Further, western blot determined that circSCAP knockdown increased the protein level of E-cadherin while diminishing that of N-cadherin, Vimentin, and MMP9 (Fig. 3F). Collectively, our results suggest that circSCAP knockdown suppresses the proliferation and metastasis of NSCLC cells.

### CircSCAP regulates SMAD2 expression by sponging miR-7

**Fig. 4:**
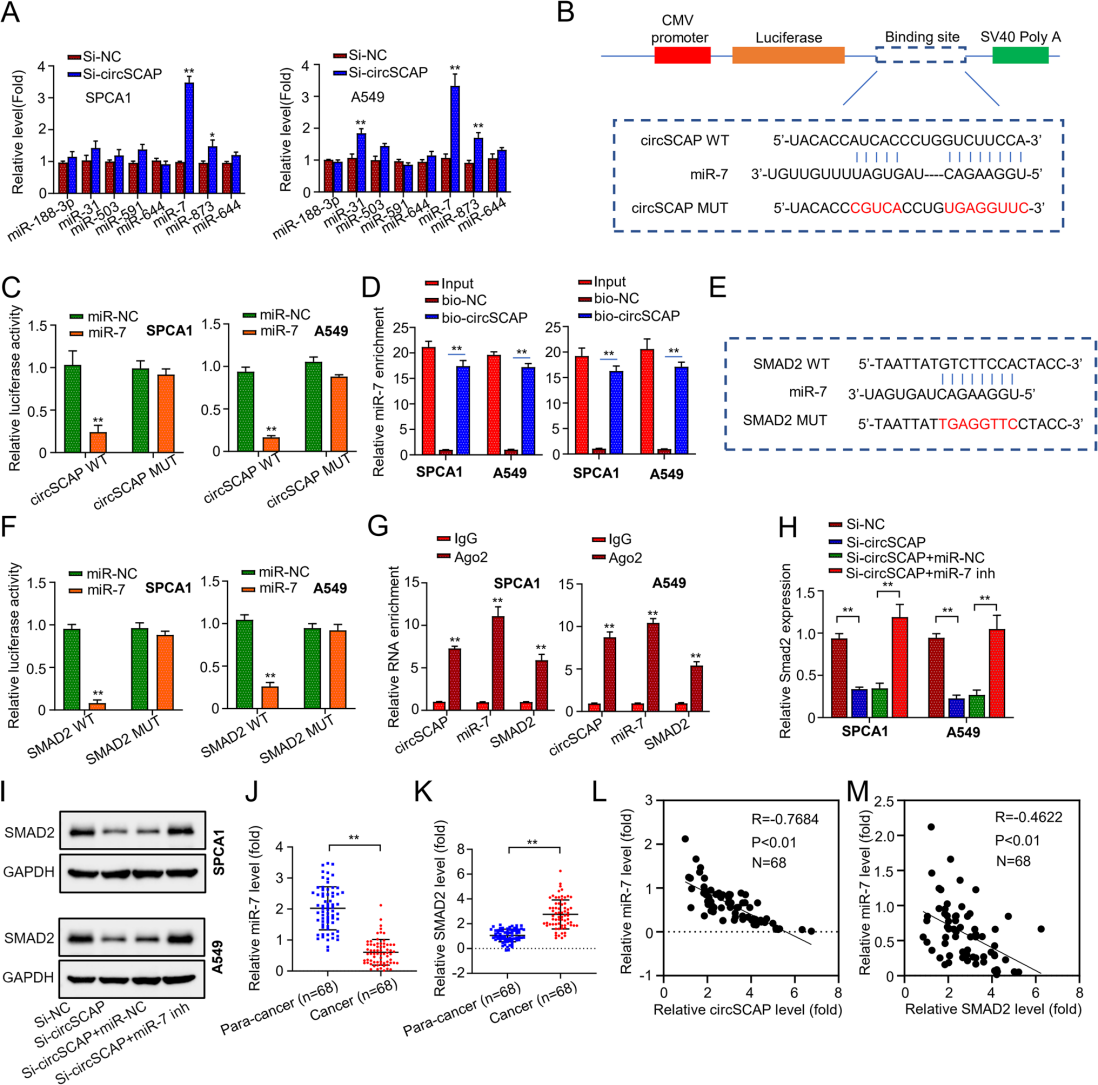
circSCAP regulates SMAD2 expression by competitively binding to miR-7. **A** Differentially expressed miRNAs inSPCA1 and A549 cells transfected with si-NC or si-circSCAP were detected by qRT‐PCR. **B** Putative circSCAP binding site in miR-7. **C** Interaction between circSCAP and miR-7 was determined by luciferase reporter assay. **D** Interaction between circSCAP and miR-7 was determined by RNA pull-down assay. **E** Complementary sequences between miR-7 and SMAD2. **F** Interaction between miR-7 and SMAD2 was determined by luciferase reporter assay. **G** Interaction between circSCAP, miR-7, and SMAD2 were determined by RNA immunoprecipitation (RIP) assay. **H** Levels of SMAD2 transcripts in SPCA1 and A549 cells transfected with indicated constructs (si-NC; si-circSCAP; si-circSCAP + inhibitor control; si-circSCAP + miR-7 inhibitor) were determined by qRT‐PCR. **I** Levels of SMAD2 protein in SPCA1 and A549 cells transfected with indicated constructs (si-NC; si-circSCAP; si-circSCAP + inhibitor control; si-circSCAP + miR-7 inhibitor) were determined by Western blot. **J** Expression levels of miR-7 in 68 pairs of NSCLC tissues and adjacent non-cancerous specimens were calculated by qRT‐PCR. **K** Levels of SMAD2 transcripts in 68 pairs of NSCLC tissues and adjacent non-cancerous specimens were calculated by qRT‐PCR. **L** Correlation between the expression of circSCAP and miR-7 in NSCLC tissues was analyzed by Pearson’s correlation coefficient. **M** Correlation between the expression of miR-7 and SMAD2 in NSCLC tissues was analyzed by Pearson’s correlation coefficient. All experiments were performed in triplicate. *P < 0.05, **P < 0.01

Next, the miRNA that was targeted by circSCAP was screened. First, we compared the expression of miR-188-3p, miR-31, miR-503, miR-591, miR-644, miR-7, miR-873, and miR-644 in SPCA1 and A549 cells transfected with si-NC or si-circSCAP using qRT-PCR. CircSCAP knockdown significantly increased the expression level of miR-7 but did not affect the expression of other miRNAs (Fig. 4A). Moreover, the bioinformatic analysis revealed that circSCAP and miR-7 could form base pairings (Fig. 4B). To confirm this interaction, we performed the luciferase reporter assay. SPCA1 and A549 cells transfected with WT-circSCAP and miR-7 mimics exhibited reduced luciferase reporter activity, whereas the cells transfected with MUT-circSCAP and miR-7 mimics did not (Fig. 4C). Further, we used the RNA pull-down assay to confirm the interaction between circSCAP and miR-7. MiR-7 was enriched in the circSCAP probe fraction (Fig. 4D), indicating that circSCAP can directly bind to miR-7.

Through the TargetScan database (https://www.targetscan.org/vert_80/), we predicted that SMAD2 was a potential target gene for miR-7 (Fig. 4E). We used the luciferase reporter assay to verify the interaction between SMAD2 and miR-7. The result revealed that co-transfection of WT-SMAD2 with miR-7 mimics reduced the luciferase reporter activity in SPCA1 and A549 cells, whereas co-transfection of MUT-SMAD2 and miR-7 did not (Fig. 4F). In addition, the RIP assay showed that Ago2 antibody precipitated Ago2 protein from the cell lysates, and circSCAP, miR-7, and SMAD2 were enriched in the Ago2 pellet (Fig. 4G), suggesting that miR-7 directly targets SMAD2 gene. Moreover, both mRNA and protein levels of SMAD2 were decreased in the cell lines with circSCAP being knocked down, and this effect was reversed by co-transfection with miR-7 inhibitors (Fig. 4H and I). In NSCLC tissues, miR-7 was downregulated, whereas SMAD2 was upregulated (Fig. 4J and K); the expression level of miR-7 was inversely correlated with that of circSCAP or SMAD2 (Fig. 4L and M). Taken together, the data indicate that circSCAP regulates SMAD2 expression by sponging miR-7.

### CircSCAP promotes NSCLC tumorigenesis and progression through regulating the miR-7/SMAD2 signaling

**Fig. 5:**
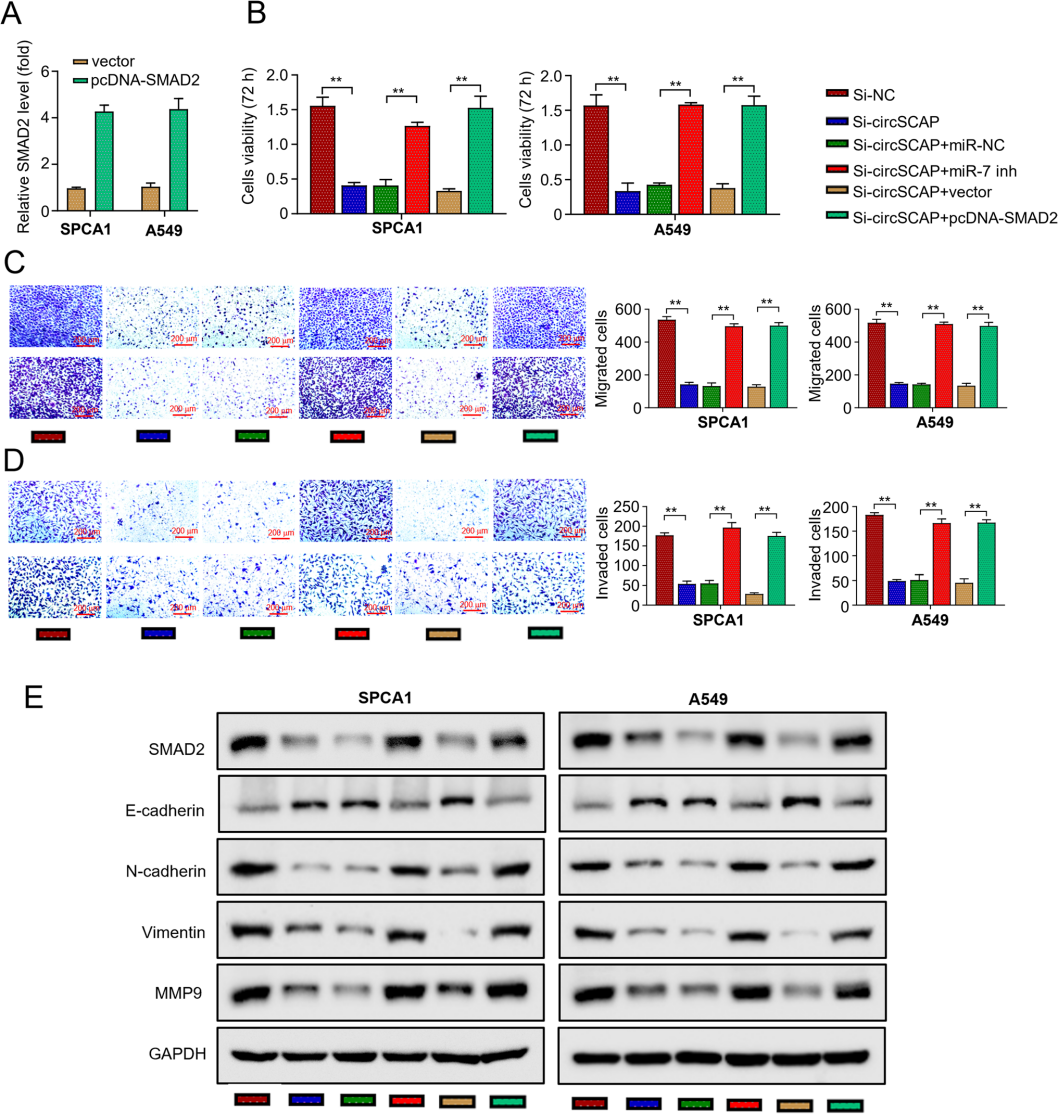
circSCAP promotes NSCLC tumorigenesis and progression through regulating the miR-7/SMAD2 signaling. **A** Levels of SMAD2 transcripts inSPCA1 and A549 cells transfected with OE-vector or OE-SMAD2 plasmids were calculated by qRT‐PCR. **B** Cell proliferation inSPCA1 and A549 cells transfected with indicated constructs (si-NC; si-circSCAP; si-circSCAP + inhibitor control; si-circSCAP + miR-7 inhibitor; si-circSCAP + OE-vector; si-circSCAP + OE-SMAD2) was determined by the CCK-8 assay. **C** Cell migration in (B) was determined by transwell assay without Matrigel coat. **D** Cell invasion in (B) was determined by transwell assay with Matrigel coat. **E** Protein levels of E-cadherin, N-cadherin, Vimentin, and MMP9 in (B) were determined through Western blot. All experiments were performed in triplicate. *P < 0.05, **P < 0.01

Given the interaction of circSCAP with miR-7 and SMAD2, we hypothesized that circSCAP promotes the tumorigenesis and progression of NSCLC by regulating the miR-7/SMAD2 axis. To test this hypothesis, we overexpressed SMAD2 in SPCA1 and A549 cells. Figure 5A reports that SMAD2 expression was significantly elevated in the cells transfected with OE-SMAD2 (Fig. 5A). As shown in Fig. 3, circSCAP knockdown inhibited the cell proliferation, migration, and invasion ability. However, this inhibiting effect could be eliminated by either inhibiting miR-7 or overexpressing SMAD2 (Fig. 5B–D). Also, the upregulation of E-cadherin and the downregulation of N-cadherin, Vimentin, and MMP9 caused by circSCAP knockdown were reversed by either inhibiting miR-7 or overexpressing SMAD2 (Fig. 5E). Collectively, these results suggest that circSCAP facilitates the proliferation and metastasis of NSCLC cells by regulating the miR-7/SMAD2 axis.

## Discussion

CircRNAs are versatile in the development and progression of malignant tumors. For instance, circRNA 001418 facilitates the invasion and metastasis of bladder carcinoma by regulating the miR-1297/EphA2 axis (Peng et al. 2021). Similarly, circ_PSD3 promotes the progression of papillary thyroid carcinoma through regulating the miR-637/HEMGN signaling (Li et al. 2021a, b). In contrast, hsa_circ_001988 suppresses the progression of gastric cancer by sponging miR-197-3p (Sun et al. 2021). In this study, through differential expression analysis of the GEO database, we found that circSCAP was significantly upregulated in NSCLC. In addition, our study revealed that circSCAP deficiency impaired the proliferation and metastasis of NSCLC cells, demonstrating the critical role of circSCAP in the tumorigenesis and progression of NSCLC. Consistently, the abnormal expression of circSCAP has been reported in NSCLC (Chen et al. 2022). In comparison, our study delineated a more detailed mechanism underlying the regulatory role of circSCAP as a ceRNA. Nevertheless, the regulation of circSCAP on NSCLC progression has been confirmed by our results and other’s.

CircRNA expression is regulated by RNA binding proteins that bind to the upstream region of pre-mRNAs (Masuda et al. 2016; Yamaguchi and Takanashi 2016; Wang et al. 2020). In the current study, we screened the CircInteractome database and predicted a putative EIF4A3 binding site in the upstream region of circSCAP pre-mRNA. RNA-binding proteins could regulate the expression of circRNAs (Beermann et al. 2016). EIF4A3 is a vital RNA splicing modulator (Chan et al. 2004). EIF4A3 induced the expression of circASAP1 and circMMP9 in glioblastoma (Wang et al. 2018a, b; Wei et al. 2021), circ_0084615 in colorectal cancer (Jiang et al. 2021), and circTOLLIP in hepatocellular carcinoma (Liu et al. 2022). Here, we confirmed the binding of EIF4A3 to circSCAP by RIP. Moreover, we found that EIF4A3 knockdown increased both mRNA and protein levels of EIF4A3 in SPCA1 and A549 cells. Collectively, we can conclude that EIF4A3 promotes circSCAP expression through binding to the upstream of circSCAP pre-mRNA.

The interactions among circRNA, miRNA, and mRNA are crucial for the pathological process of malignant tumors (Rong et al. 2017; Xiong et al. 2018; Liang et al. 2020). MiRNAs are highly conserved (Thomson and Dinger 2016) and typically act as ceRNAs to regulate target gene expression (Salmena et al. 2011). On the other hand, miRNA expression is dynamically regulated by circRNAs (Cheng et al. 2019; Zhang et al. 2020a, b; Shi et al. 2021). This hierarchical regulatory axis modulates the progression of diverse malignancies including NSCLC. In this study, we identified miR-7 as the target for circSCAP. In NSCLC tissues, miR-7 expression was significantly decreased and was inversely correlated with circSCAP expression. All the data indicate that miR-7 is sponged by circSCAP in NSCLC.

MiRNAs regulate cell activities by suppressing target gene expression (Saliminejad et al. 2019). In this study, using TargetScan screening, the luciferase reporter assay, and RIP, we found small mothers against decapentaplegic 2 (SMAD2) as the target gene of miR-7. SMAD2 acts as a downstream effector in the TGF-β signaling pathway and is essential for pattern formation and tissue differentiation (Massague 2012; Hata and Chen 2016; Granadillo et al. 2018). The degradation of SMAD2 transcripts mediated by miRNAs contributes to tumorigenesis (Hu et al. 2017; Zhang et al. 2019a, b; Shi et al. 2020). More interestingly, SMAD2 has been shown to play a key role in NSCLC. For instance, SMAD2 expression was upregulated by lncRNA OSER1‑AS1 through sponging miR‑433‑3p, which promotes the malignant properties of NSCLC (Liu et al. 2020). Similarly, our work showed that SMAD2 expression was elevated and inversely correlated with miR-7 expression in NSCLC tissues. Further, our mechanistic investigation revealed that circSCAP deficiency undermined SMAD2 expression in NSCLC cells, which could be rescued by co-transfection with miR-7 inhibitors. These studies indicate that SMAD2 expression is tightly regulated by miR-7 in NSCLC.

In conclusion, this study demonstrates that circSCAP is upregulated in NSCLC cells and facilitates NSCLC progression by sponging miR-7 and upregulating SMAD2, providing a new perspective for early diagnosis and treatment of NSCLC. Whether circSCAP promotes the progression of other types of cancers through the same signaling awaits further studies.

## Data Availability

The datasets used and/or analyzed during the current study are available from the corresponding author on reasonable request.
